# GlideScope® versus C-MAC®(D) videolaryngoscope versus Macintosh laryngoscope for double lumen endotracheal intubation in patients with predicted normal airways: a randomized, controlled, prospective trial

**DOI:** 10.1186/s12871-020-01012-y

**Published:** 2020-05-20

**Authors:** Ping Huang, Renlong Zhou, Zhixing Lu, Yannan Hang, Shanjuan Wang, Zhenling Huang

**Affiliations:** Department of Anesthesiology, Renji Hospital, School of Medicine, Shanghai Jiaotong University, Shanghai, 200001 China

**Keywords:** GlideScope® videolaryngoscope, C-MAC®(D) videolaryngoscope, Macintosh laryngoscope, Double lumen tracheal tube, Endotracheal intubation

## Abstract

**Background:**

The double lumen endotracheal tube (DLT) is the most widely-used device for single lung ventilation in current thoracic anesthesia practice. In recent years, the routine application of the videolaryngoscope for single lumen endotracheal intubation has increased; nevertheless there are few studies of the use of the videolaryngoscope for DLT. We wondered whether there were benefits to using the videolaryngoscope for DLT placement in patients with predicted normal airways. Therefore, this study was designed to compare the performances of the GlideScope®, the C-MAC®(D) videolaryngoscope and the Macintosh laryngoscope in DLT intubation.

**Methods:**

This was a randomized, controlled, prospective study. We randomly allocated 90 adult patients with predicted normal airways into three groups. All patients underwent routine anesthesia using different laryngoscopes according to group allocation. We compared DLT insertion times, first-pass success rates, numerical rating scales (NRS) of DLT delivery and DLT insertion, Cormack-Lehane degrees (C/L), hemodynamic changes and incidences of intubation complications. All outcomes were analyzed using SPSS13.0.

**Results:**

Compared with the GlideScope, the Macintosh gave shorter times for DLT insertion (median: 96 (IQR: 51 [min–max: 62–376] s vs 73 (26 [48–419] s, *p* = 0.003); however, there was no difference between the Macintosh and C-MAC(D) (*p* = 0.610). The Macintosh had a significantly higher successful first attempt rate than did the GlideScope or C-MAC(D) (*p* = 0.001, *p* = 0.028, respectively). NRS of DLT delivery and insertion were significantly lower in the Macintosh than in the others (*p* < 0.001). However, the C/L degree in the Macintosh was significantly higher than in the others (p < 0.001). The incidences of oral bleeding, hoarseness, sore throat and dental trauma were low in all groups (*p* > 0.05). There were no significant differences in DLT misplacement, fiberoptic time or hemodynamic changes among the groups.

**Conclusions:**

Compared with the Macintosh laryngoscope, the GlideScope® and C-MAC®(D) videolaryngoscopes may not be recommended as the first choice for routine DLT intubation in patients with predicted normal airways.

**Trial registration:**

The study was prospectively registered at the Chinese Clinical Trial Registry (no. ChiCTR1900025718); principal investigator: Z.L.H.; date of registration: September 6, 2019.

## Background

The double lumen endotracheal (DLT) tube is the most widely-used device for single lung ventilation in current thoracic anesthesia practice [1]. Nevertheless, on account of its relatively large diameter, larger volume of oral cavity occupation, and rotating insertion technique, the DLT is generally more difficult to insert and advance than is the single lumen endotracheal tube.

In recent years, videolaryngoscopes have become the new standard of care for intubation because they provide clear views of the glottis using a video-camera or video-chip that is positioned close to the tip of the laryngoscope blade. Each available videolaryngoscope is unique in terms of design [2, 3]. They can be divided into three types according to blade type: the standard Macintosh-shaped blade, the angulated blade, and a channel for tube passage [4].

The GlideScope® is a videolaryngoscope with a highly-angulated blade form that offers an obligatory indirect view of the epiglottis [5]. Several authors described use of the GlideScope® in patients with difficult airways during anesthesia [6–9]. Most authors found that the GlideScope® gave better laryngoscopic views than did either conventional direct laryngoscopy or Macintosh videolaryngoscopy in patients with predicted difficult airways [5]. Serocki et al. concluded that the GlideScope® and the C-MAC® may be useful in the management of predicted difficult airways [6]. Stroumpoulis et al. suggested that it could be a logical alternative in the management of difficult airways for single-lumen endotracheal intubation [7]. Sun et al. found that the GlideScope® provided laryngoscopic views equal to or better than those of direct laryngoscopy in 200 patients; however, the device required additional 16 s to achieve tracheal intubation [8]. In a systematic review and meta-analysis, Donald et al. found that, compared to direct laryngoscopy, Glidescope® videolaryngoscopy was associated with improved glottic visualization, particularly in patients with potential or simulated difficult airways [9].

The C-MAC® videolaryngoscope was introduced with conventional Macintosh blades, and has been used appropriately for routine airway intubation [10]. To manage difficult airways, the C-MAC® system recently introduced the highly angulated D-Blade [11]. The GlideScope® and C-MAC®(D) videolaryngoscope are generally of the same type, possessing angulated blades. They are the only videolaryngoscopes available in anesthesiology departments in China.

There are a few studies investigating angulated videolaryngoscopes for DLT intubation. The potential advantages of the GlideScope® for DLT insertion include a better view of the vocal cords, a clear view of the DLT when it passes the vocal cords, and an external video screen for teaching purposes and for the assistant providing external laryngeal pressure [12]. Nevertheless, at present, it remains controversial as to whether the GlideScope® videolaryngoscope possesses advantages for double lumen endotracheal intubation [12, 13]; to our knowledges, there has been only one article reporting the performance of the C-MAC®(D) for double lumen endotracheal intubation [14]. Therefore, in the present study, we compared the performances of the GlideScope®, C-MAC®(D) videolaryngoscope and the Macintosh laryngoscope in DLT intubation in normal airways.

## Methods

Approval for the study was granted by the Shanghai Renji Hospital Ethics Committee (Ethical number: 2016[036]). Written informed consent was obtained from patients undergoing elective intra-thoracic surgeries requiring double lumen intubation. The present trial was registered at http://www.chictr.org.cn (registration number ChiCTR1900025718; principal investigator: Z.L.H.; date of registration: September 6, 2019).

Inclusion criteria were as follows: age 18–75 years old; ASA I–II, BMI < 35 kg/m^2^, with Mallampati score of 1 or 2. All Mallampati scores were assigned by the same observer. Exclusion criteria were as follows: presence of any predictors of difficult intubation; Mallampati score > =3; inter-incisor distance < 3 cm; thyromental distance < 6 cm; neck extension < 80°from neck flexion; cervical spine instability; history of difficult endotracheal intubation or difficult mask ventilation; and severe pulmonary ventilation dysfunction or risk of pulmonary aspiration.

**Fig. 1:**
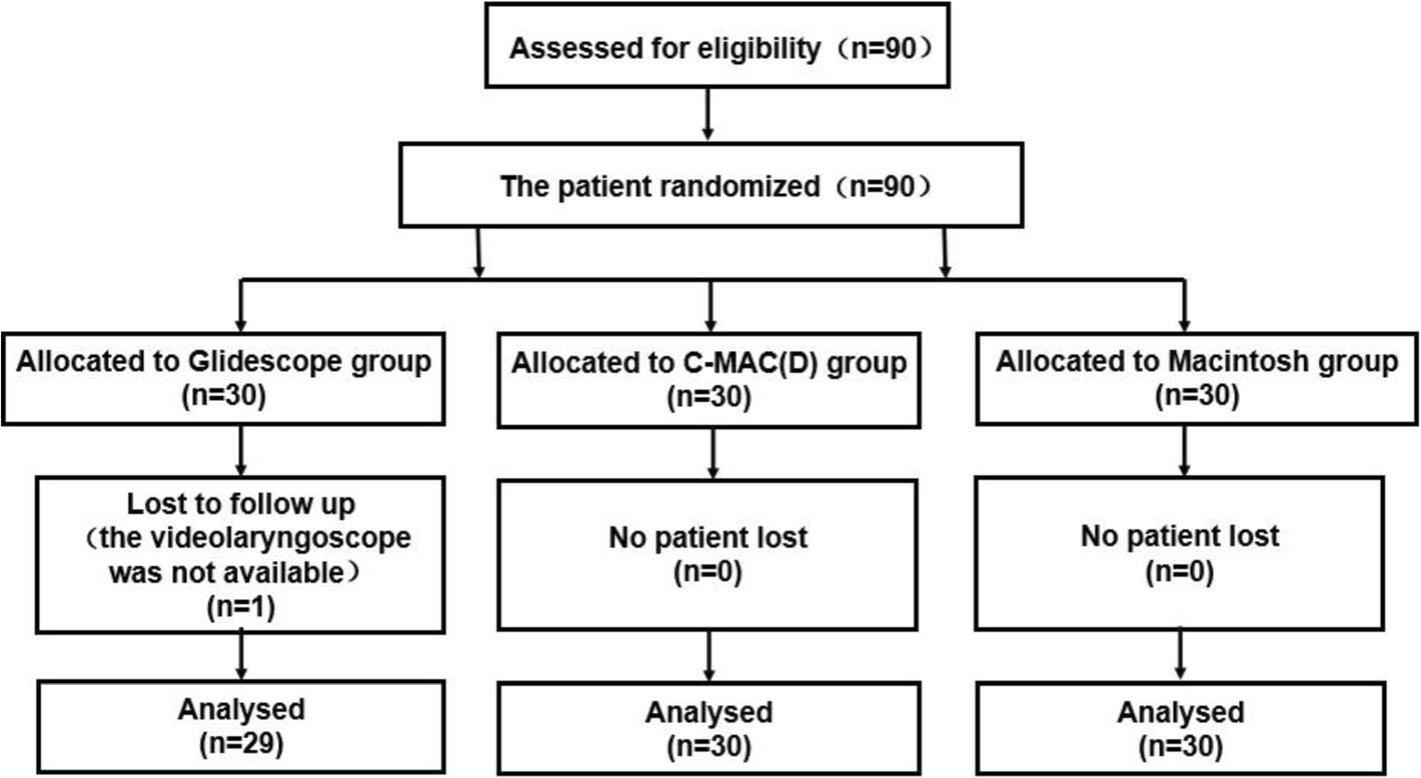
CONSORT flow diagram of patient selection and allocation

Eligible patients were enrolled on the basis of the CONSORT Statement Extension for Randomized Controlled Trials of Nonpharmacological Trials, as displayed in the flowchart in Fig. 1. We randomly assigned 90 patients to GlideScope (Verathon Medical, Bothwell, UT, USA), C-MAC(D) (Karl Storz GmbHand Co.KG, Tuttlingen, Germany), or Macintosh groups. This was done using a closed envelope technique using a computer-generated block randomization method in blocks of 15. Before the study, the computerized randomization was performed and the allocation results were placed in individual numbered and sealed envelopes. The researcher responsible for recruitment blinded to the allocation result. After a patient was consented for the study, allocation was revealed. All endotracheal intubations were performed by five anesthesiologists with 10 years’ working experience skilled in videolaryngoscopy.

Left-side or right-side 32Fr/35Fr Mallinckrodt™ DLTs (Mallinckrodt Medical, Athlone, Ireland) were selected for female patients and 35Fr/37Fr DLTs for male patients depending on whether their heights were below or above 155 cm for females and 165 cm for males. If the operation side was the left, right-side DLT was used; otherwise, the left-side DLT was used. To facilitate intubation, the distal 10–12 cm concavity of the DLT (with the stylet in situ) was molded along the blade convexity in each group. The tracheal and the bronchial cuffs of the DLT tubes were lubricated with sterile Surgilube.

No premedication was given before induction. Standard monitoring prior to induction included ECG, invasive arterial blood pressure, SpO_2_, and end-tidal carbon dioxide. After pre-oxygenation with 100% oxygen, anesthesia was induced with intravenous midazolam 0.05 mg^.^kg^− 1^, propofol 1.5 mg^.^kg^− 1^, fentanyl 5 μg^.^kg^− 1^, and rocuronium 0.6 mg^.^kg^− 1^. Ninety seconds after rocuronium administration, DLT intubation was performed using the allocated laryngoscope. The DLT was inserted with the distal concavity facing anteriorly until the bronchial lumen cuff passed the vocal cords. The stylet was then removed, and rotation was performed while tube was advanced. The left DLT rotated 90° counter-clockwise, and the right DLT was rotated 90° clockwise to enter the respective mainstem bronchus. Hemodynamic changes were monitored during induction. If systolic blood pressure fell below 80 mmHg, ephedrine 5 mg was administrated intravenously. Atropine 0.5 mg was given for heart rate below 50 beats per minute. After the tip of the DLT was located in the targeted bronchus, the tracheal cuff was inflated and ventilation of the lungs started. Fiberoptic bronchoscopic assessment of adequate bronchial cuff placement was followed by DLT placement.

DLT insertion time was defined as from the time the laryngoscope passed the patient’s lips until three complete end-tidal carbon dioxide cycles were displayed on the monitor. Intubation success rate at the first attempt was recorded by the same observer. The difficulty of DLT insertion and delivery were assessed by the operator, using NRS ranging from 0 to 10. The NRS results were grouped as 0 = none, 1–3 = mild, 4–6 = moderate, and 7–10 = severe. C/L degrees were classified as four degrees (I, II_A_, II_B_, and III) and were assessed by the same operator. If the degree was not class I, external laryngeal pressure was provided by an assistant. The time taken for fiberoptic bronchoscopy was defined as the time from endobronchial intubation to placement confirmation using fiberoptic bronchoscopy. The operators examined blade surfaces for blood after removal. Hemodynamic parameters (mean arterial blood pressure and heart rate) were recorded 10 min before induction and 1, 3, and 5 min after intubation. After the assessment by fiberoptic bronchoscopy, the oral cavity, pharynx, larynx and teeth were examined for signs of laceration or bleeding by an independent investigator who was unaware of the type of laryngoscope used. One day after surgery, an independent investigator interviewed patients to assess the presence of sore throat and hoarseness of voice.

### Statistical analysis

Based on previous studies [13, 14], we determined that the mean intubation time for the GlideScope would be 45.6 s with a standard deviation of 10.7 s, and that of the C-MAC(D) would be 32.27 s with a standard deviation of 11.13 s [12]. Factoring possible drop-outs, we recruited 30 patients in each group, with an alpha value of 0.05 and a beta value of 0.2.

Data were expressed as median (interquartile range (IQR) [min–max]), mean ± SD, or absolute numbers, as required. Statistical analysis was performed using SPSS 13.0 (SPSS Inc., Chicago, IL). The Kruskal–Wallis test was used to analyze independent samples (the success rate at the first attempt, the times of intubation attempts, the DLT insertion time, the number of external laryngeal pressure applications, C/L degree, and NRS of DLT delivery and insertion). The Chi-square and Student–Newman–Keuls tests were used to analyze demographic data and the incidence of complications. For the analysis of hemodynamic response to intubation, a repeated-measures analysis of variance was used. Statistical significance was considered at *P* < 0.05.

## Results

The CONSORT flow diagram of the study is shown in Fig. 1. Of the 90 patients recruited, 89 completed the study. One patient in the GlideScope group was excluded because the videolaryngoscope was not available. Characteristics of patients and intubation conditions were similar in all three groups (Table 1).

**Table 1:** Characteristics and intubation conditions of patients assigned to GlideScope, C-MAC(D) or Macintosh group

	GlideScope group (*n* = 29)	C-MAC(D) group (*n* = 30)	Macintosh group (*n* = 30)
Age (yr)	58.45 ± 8.80	57.20 ± 9.60	54.57 ± 11.78
BMI (kg/m^2^)	23.33 ± 3.29	22.82 ± 2.67	24.32 ± 3.78
Male/Female (n)	11/18	18/12	20/10
ASA I/II (n)	17/12	18/12	20/10
DLT left/right (n)	17/12	21/9	21/9
Malampati I/II (n)	17/11	17/13	19/11
Inter–incisor distance (cm)	4.29 ± 0.60	4.43 ± 0.77	4.53 ± 0.88
Thyromental distance (cm)	7.77 ± 0.78	7.97 ± 0.79	7.72 ± 0.75
Neck extension> 90^0^ (n)	29	30	30

**Fig. 2:**
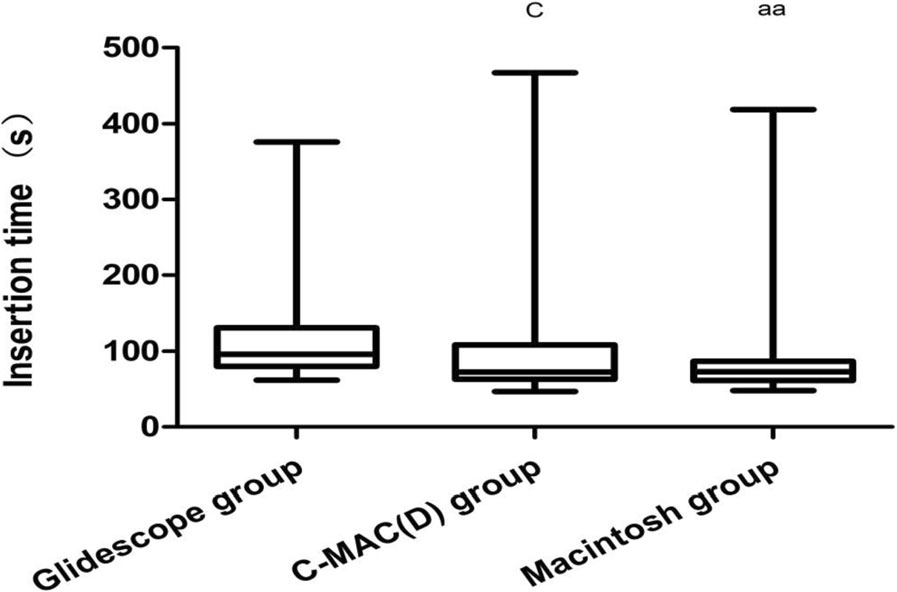
The time taken for bronchial insertion with GlideScope was significantly longer compared with those taken for Macintosh and C-MAC(D). GlideScope vs C-MAC(D) vs Macintosh: 96 s (51 [62–376] s) vs 72.5 s (46 [47–467] s) vs 73 s (26 [48–419] s); ^aa^*P* < 0.01, between the GlideScope and Macintosh groups. ^c^*P* < 0.05, between the GlideScope and C-MAC(D) groups

The median DLT insertion time was 96 s (51 [62–376] s) with the GlideScope, 73 s (26 [48–419] s) with the Macintosh (*p* = 0.003) and 72.5 s (46 [47–467] s) with the C-MAC(D) (*p* = 0.022). There was no difference between the Macintosh and C-MAC(D) (*p* = 0.610) (Fig. 2).

The number of successes at the first attempt was 24 (80%) with the Macintosh, 11 (38%) with the GlideScope (*p* = 0.001), and 16 (53%) with the C-MAC(D) (*p* = 0.028). There was no difference between the GlideScope and the C-MAC(D) (*p* = 0.235) (Table 2).

**Table 2:** Details of intubation with a double-lumen tube using the GlideScope, C-MAC(D) or Macintosh group

	GlideScope group(*n* = 29)	C-MAC(D) group(*n* = 30)	Macintosh group(i = 30)	*P* value
Number of intubation attempts (1/2/3)	11/12/6	16/11/3	24/3/3 ^aab^	0.010
The success at the first attempt (n)	11 (37.93%)	16 (53.33%)	24 (80.00%) ^aab^	0.004
Cormack-Lehane degree(I/II_A_/II_B_/III)	14/14/1/0 ^cc^	26/4/0/0	9/9/5/7 ^aabb^	0.000
Number of external laryngeal pressure (n)	4 (13.79%) ^c^	0 (0)	14 (46.67%) ^aabb^	0.000
Number of DLT misplacement (n)	4 (13.79%)	9 (30.00%)	5 (16.67%)	0.252
Fibreoptic time (s)	54.07 ± 25.46	63.13 ± 23.77	57.43 ± 21.8	0.309
Oral bleeding (n)	1 (3.45%)	1 (3.33%)	0 (0)	0.594
Hoarseness (n)	0 (0)	0 (0)	1 (3.33%)	0.370
Sore throat (n)	1 (3.45%)	3 (10%)	1 (3.33%)	0.441
Dental trauma (n)	0 (0)	0 (0)	0 (0)	
Atropine (n)	0 (0)	0 (0)	0 (0)	
Ephedrine (n)	1 (3.45%)	1 (3.33%)	0 (0)	0.594

^aa^p < 0.01 between Glidescope and Macintosh

^bb^p < 0.01 between C-MAC(D) and Macintosh

^b^p < 0.05 between C-MAC(D) and Macintosh

^cc^*p* < 0.01 between Glidescope and C-MAC(D)

^c^*p* < 0.05 between Glidescope and C-MAC(D)

**Fig. 3:**
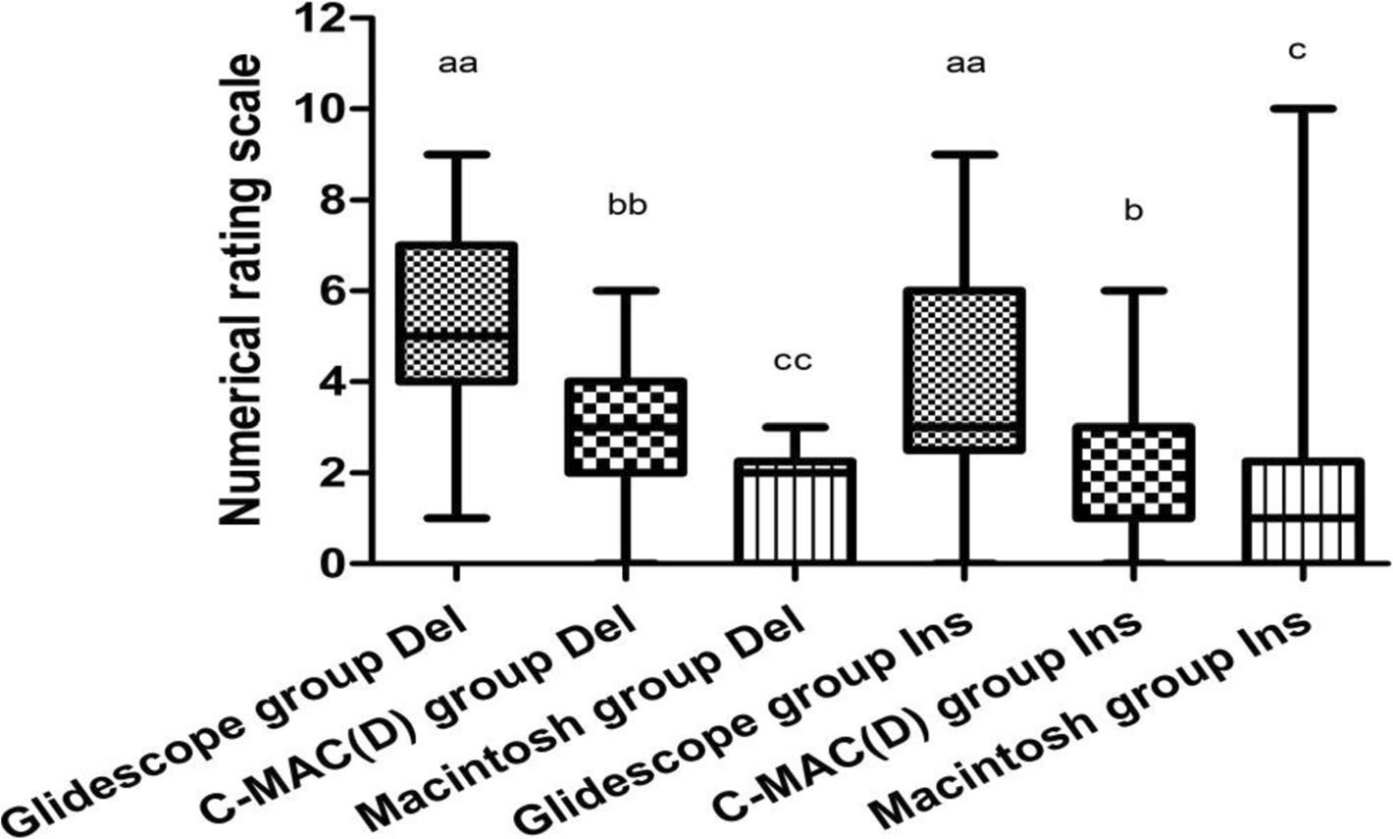
Del and Ins represent Delivery and Insertion. As for NRS (scored from 0 to 10), difficulty score of DLT delivery was 5 (3 [1–9]) with the GlideScope, 3 (2 [0–6]) with the C-MAC(D) (p = 0.000), 2 (1.75 [0–3]) with the Macintosh (*p* = 0.000). C-MAC(D) vs the Macintosh (p = 0.001). Difficulty score of DLT insertion was 3 (3 [0–9]) with the GlideScope, 3 (1.75 [0–6]) with C-MAC(D) (*p* = 0.039) and 1 (2 [0–10]) with Macintosh (*p* = 0.001). C-MAC(D) vs Macintosh *p* = 0.026. ^aa^*P* < 0.01, between the GlideScope and Macintosh groups; ^bb^P < 0.01, between the C-MAC(D) and Macintosh groups; ^b^*P* < 0.05, between the GlideScope and C-MAC(D) groups; ^cc^P < 0.01, between the C-MAC(D) and Macintosh groups; ^c^P < 0.05, between the C-MAC(D) and Macintosh groups

The median NRS of DLT delivery was 2 (1.75 [0–3]) with the Macintosh, 5 (3 [1–9]) with the GlideScope (*p* = 0.000), and 3 (2 [0–6]) with the C-MAC(D) (p = 0.001). The *P*-value for the difference between the GlideScope and the C-MAC(D) was 0.000. The median NRS of DLT insertion was 1 (2 [0–10]) with the Macintosh, 3 (3 [0–9]) with the GlideScope (p = 0.001), and 3 (1.75 [0–6]) with the C-MAC(D) (*p* = 0.026). The P-value for the difference between the GlideScope and the C-MAC(D) was 0.039 (Fig. 3).

C/L degrees (I/II_A_/II_B_/III) were 14/14/1/0 with the GlideScope, 26/4/0/0 with the C-MAC (D) (*p* = 0.006), and 9/9/5/7 with the Macintosh (*p* = 0.008). The P-value for the difference between the C-MAC(D) and the Macintosh was 0.000. The data indicate that external laryngeal pressure was used most often in the Macintosh group (Table 2).

There were no differences in the number of DLT misplacements or fiberoptic times. Blood was observed on the laryngoscopes in only one patient in each of the GlideScope and the C-MAC(D) groups. We found a low incidence of oral bleeding, sore throat, and dental trauma in all three groups, with no significant differences. One patient each required ephedrine in the GlideScope and C-MAC(D) groups; however, no patient in any group required atropine (Table 2).

**Fig. 4:**
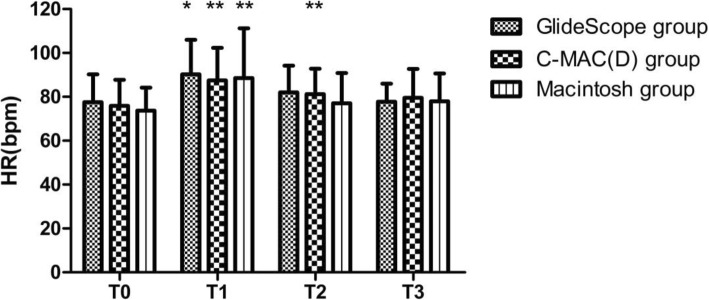
T0 represents the basic heart rate 10 min before the study. T1, T2 and T3 represent 1 min, 3 min and 5 min after intubation, respectively. Heart rate increased 1 min after intubation in all three groups and increased 3 min after intubation in the C-MAC(D) group. Compared with T0, GlideScope *P < 0.05, C-MAC(D) **P < 0.01, Macintosh **P < 0.01 in T1, C-MAC(D) *P* < 0.01 in T2

**Fig. 5:**
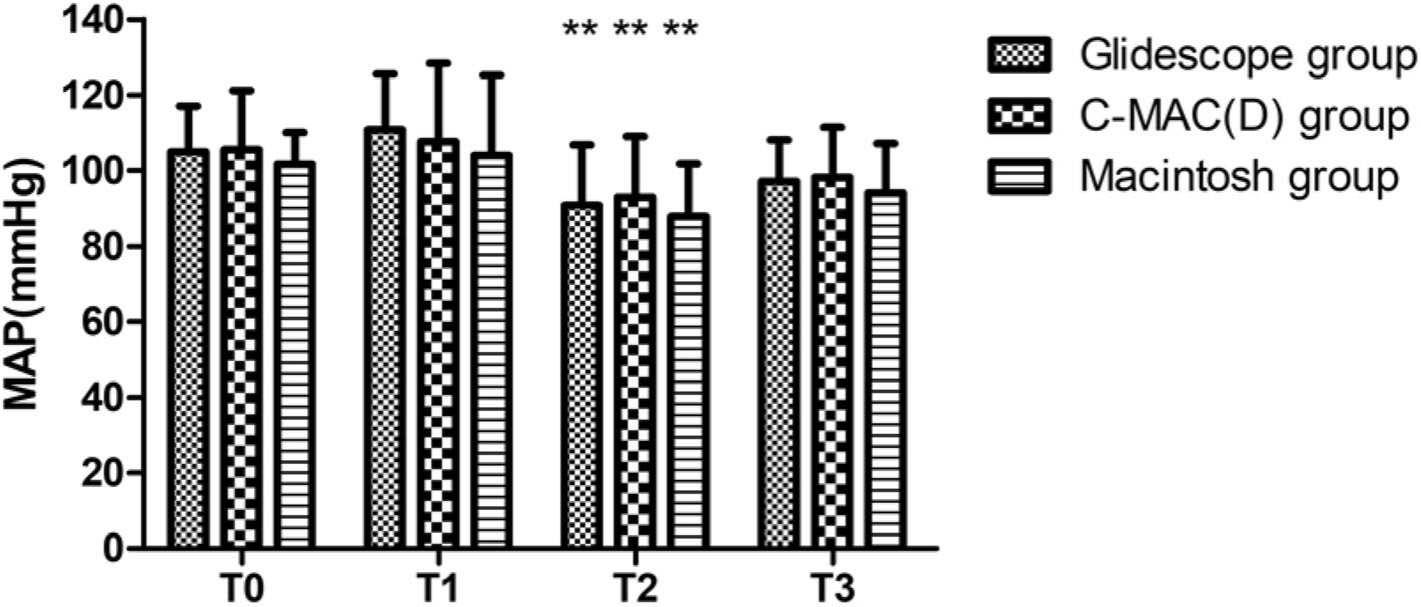
T0 represents the basic mean arterial pressure 10 min before the study. T1, T2 and T3 represent 1 min, 3 min and 5 min after intubation, respectively. Mean arterial pressure decreased 3 min after intubation in all three groups. Compared with T0, three groups **P < 0.01 in T2

Heart rate increased 1 min after intubation in all three groups and increased 3 min after intubation in the C-MAC(D) group (Fig. 4). Mean arterial pressure decreased 3 min after intubation in all three groups (Fig. 5). There was no difference between groups with respect to hemodynamic responses to intubation.

## Discussion

In our study comparing the GlideScope® and C-MAC®(D) videolaryngoscopes with the Macintosh laryngoscope to assist DLT intubation, we found that the Macintosh was associated with shorter insertion time, higher success rate at the first attempt, less difficulty score of DLT delivery and insertion, higher C/L degree and more use of external laryngeal pressure. To the best of our knowledge, no study has compared the GlideScope® and C-MAC®(D) videolaryngoscopes with the Macintosh laryngoscope for DLT intubation, highlighting the novelty of the present trial.

The GlideScope® videolaryngoscope was the first obligatory indirect videolaryngoscope with a pronounced anterior angulation of 60° of its blade [5]. The C-MAC®(D) videolaryngoscope has a pronounced angulation of 40°. In contrast to the GlideScope®, the D-Blade’s camera and light socket are located closer (40 mm) to the blade’s tip, which is bent another 20° [15].

With the attached cables and the angulated blade form, the GlideScope® and C-MAC®(D) videolaryngoscopes often require more delicate manipulation than the conventional Macintosh laryngoscope when they are introduced into the mouth and advanced further. Nevertheless, manipulation inside the oropharyngeal space is restricted because of the high degree of blade angulation. When proceeding in a relatively steep angle through the glottic opening, the DLT may get caught at the arytenoids or the ventral tracheal wall. The use of both videolaryngoscopes requires hand-eye coordination on the part of the operator. The operator should identify the glottis and vocal cords on the screen, then manipulate the DLT to enter into the mouth and past the vocal cords into trachea; doing so may prolong insertion time. Videolaryngoscopes with large bending blades often require larger angles of the distal stylet to enable the tracheal tube to reach the glottis through the “corner field” of the blade [16]. This increases the difficulty of tube delivery and insertion time. Nevertheless, in the present study, the insertion time of the C-MAC(D) was similar to that of the Macintosh. The reason may be that both blades are relatively thin. This increased room in the oral cavity for intubation and made the double lumen canal rotation relatively convenient. Second, because the images obtained by C-MAC®(D) videolaryngoscope include the tip of the blade, the device can be placed in the epiglottic valley under monitor vision. This also shortens insertion time. Because of the large outer diameter and more rigid design of DLTs, they are relatively harder to insert alongside the angulated and thick blade of the GlideScope®, which necessitates angulation of the tip of the DLT to follow the curve of its angulated blade, together with sequential rotation of the DLT. This potentially could prolong the time required for DLT insertion [17].

The advantages of the GlideScope® and C-MAC®(D) videolaryngoscope for DLT insertion include the following: improved views of vocal cords and of the DLT when the devices pass the vocal cords; external video monitors for assisting staff who apply external pressure to the larynx; and finally, these devices are ideal for teaching purposes. Their disadvantages include increased blade angulation and thickness that may cause difficulty in manipulating the DLT to enter into the mouth and pass the vocal cords into the trachea [12]. This decreases the success rate of the first attempt. At the same time, forward movement of the videolaryngoscope lens and requirement for hand-eye coordination on the part of the operator decrease the success rate at the first attempt. Although the Macintosh laryngoscope showed a higher C/L degree, that is unfavorable to endotracheal intubation, glottic views were improved by application of external laryngeal pressure. These data suggest that the Macintosh laryngoscope had a higher success rate at the first attempt.

Due to the thicker blade of GlideScope® and the larger DLT diameter, difficulty with DLT manipulation and difficulty in fitting the device and entering into patient’s mouth were the most common reasons for GlideScope intubation failures, as previously reported [12]. Blades with large cambers and forward movement of the C-MAC®(D) lens increased difficulty of DLT delivery and insertion. DLT delivery and insertion scores were lower with the Macintosh than with the GlideScope or C-MAC(D). Our findings support the common view that tube delivery and advancement into the trachea are the most difficult steps when using non-channeled hyper-angulated videolaryngoscopes [16].

There was no difference between left or right DLT with respect to the incidence of the DLT entering the wrong bronchus among the groups, suggesting that misplacement had no obvious association with any laryngoscope. The original preformed shape of the anterior concavity of the DLT was slightly altered to conform to the greater angulation of the D-blade and the GlideScope before intubation, possibly increasing DLT misplacement. Nevertheless, this could be easily rectified using the fiberoptic bronchoscopy.

Postoperative sore throat and hoarseness are common complications after using DLT [18]; nevertheless, we found a low incidence of oral bleeding, hoarseness, sore throat and dental trauma in all three groups. This may be the case for two reasons. First, the DLT tubes were adequately lubricated and operated by experienced anesthesiologists. Second, 90° rotation aligned the axis of the tracheal lumen with the patient’s tracheal axis. This facilitated the passage of the tracheal cuff through the vocal cords and reduced the incidence of vocal cord injury. Hsu et al. described the method to rotate the DLT counterclockwise 180° to facilitate passage of the bronchial cuff. After the tracheal cuff passed through vocal cords, an additional 90° clockwise rotation was performed to align the tube with the left main bronchus [13]. The number of rotations was less in our study than in Hsu’s; this may explain the lower incidence of hoarseness and sore throat. There were no differences in hemodynamic changes among the groups, suggesting that videolaryngoscopy did not reduce the stress response caused by DLT intubation.

### Limitations

There are a few limitations in our study. First, the intubating anesthesiologist or independent observer was unblinded to the randomization of the videolaryngoscope. This could lead to bias. Nevertheless, the primary outcome and most of the other outcomes were objective and well-defined. Second, the operators were five anesthesiologists who had substantial experience of using the GlideScope® and the C-MAC®(D) videolaryngoscopes as well as practical techniques of DLT for administration of thoracic anesthesia. This study excluded young anesthesiologists who were inexperienced; this may have been another source of bias. Third, recruited patients had normal airways. It is not possible to comment as to whether these findings would be consistent with DLT intubation of difficult airways. Such a study should be performed in the future. Finally, we did not include videolaryngoscopes with standard Macintosh shaped blades.

## Conclusion

Compared with the Macintosh laryngoscope, the GlideScope® and C-MAC®(D) videolaryngoscopes may not be recommended as the first choice for routine DLT intubation in patients with predicted normal airways.

## Data Availability

The datasets generated during the current study are not publicly available due the regulation of data management of Renji Hospital, School of Medicine, Shanghai Jiaotong University, but are available from the corresponding author on reasonable request.

## Abbreviations

DLT: Double lumen endotracheal tube
NRS: Numerical rating scale
C/L degree: Cormack-Lehane degree
ASA: American Society of Anesthesiologists
BMI: Body mass index
ECG: Electrocardiogram
SpO_2_: Pulse oximetry saturation
